# Operational challenges in conducting a community-based technology-enabled mental health services delivery model for rural India: Experiences from the SMART Mental Health Project

**DOI:** 10.12688/wellcomeopenres.14524.1

**Published:** 2018-04-18

**Authors:** Pallab K. Maulik, Sudha Kallakuri, Siddhardha Devarapalli

**Affiliations:** 1 George Institute for Global Health India, New Delhi , 110025, India; 2 Faculty of Medicine, University of New South Wales, Sydney, NSW, 2052, Australia; 3 George Institute for Global Health, University of Oxford, Oxford, OX1 3QX, UK

**Keywords:** mental health, rural, community based services, technology enabled solutions, India

## Abstract

**Background: **There are large gaps in the delivery of mental health care in low- and middle-income countries such as India, and the problems are even more acute in rural settings due to lack of resources, remoteness, and lack of infrastructure, amongst other factors. The Systematic Medical Appraisal Referral and Treatment (SMART) Mental Health Project was conceived as a mental health services delivery model using technology-based solutions for rural India. This paper reports on the operational strategies used to facilitate the implementation of the intervention.

**Method:** Key components of the SMART Mental Health Project included delivering an anti-stigma campaign, training of primary health workers in screening, diagnosing and managing stress, depression and increased suicide risk and task sharing of responsibilities in delivering care; and using mobile technology based electronic decision support systems to support delivery of algorithm based care for such disorders. The intervention was conducted in 42 villages across two sites in the state of Andhra Pradesh in south India. A pre-post mixed methods evaluation was done, and in this paper operational challenges are reported.

**Results:** Both quantitative and qualitative results from the evaluation from one site covering about 5000 adults showed that the intervention was feasible and acceptable, and initial results indicated that it was beneficial in increasing access to mental health care and reducing depression and anxiety symptoms. A number of strategies were initiated in response to operational challenges to ensure smoother conduct of the project and facilitated the project to be delivered as envisaged.

**Conclusions:** The operational strategies initiated for this project were successful in ensuring the delivery of the intervention. Those, coupled with other more systematic processes have informed the researchers to understand key processes that need to be in place to develop a more robust study, that could eventually be scaled up.

## Introduction

Low and middle-income countries (LMICs) such as India are witnessing a rising burden of mental disorders ^1^ and a widening gap in fulfilling the expected treatment needs of those needing care (treatment gap), due to lack of adequate mental health professionals, poor infrastructure and policies related to mental health care, stigma related to help-seeking for mental illness, and general attitude of the community towards mental health ^2, 3^. This treatment gap is more among rural communities where there are even fewer resources compared to urban settings and one needs to develop strategies to bridge that gap effectively. One such solution could be using technology-enabled mental health services delivery platforms. The Systematic Medical Appraisal Referral and Treatment (SMART) Mental Health Project was conceived as such, and a technology-enabled mental health services delivery model along with an anti-stigma campaign was rolled out across rural communities ^4^. This paper outlines some key operational challenges faced while conducting the study and measures taken to overcome them.

## Methods

### The SMART Mental Health Project background

The SMART Mental Health Project was conducted across 42 villages in West Godavari district of the southern Indian state of Andhra Pradesh. In total, 30 of the villages were from a predominantly tribal area and the remining 12 were from non-tribal villages. Primary health workers in villages - both lay village health workers called Accredited Social Health Activists (ASHAs) and primary health care doctors – were trained to screen, diagnose and treat patients suffering from stress, depression, and increased suicidal risk using validated screening and diagnostic tools, that were implemented using tablet based electronic decision support systems (EDSS) ^5^. In addition, there was an anti-stigma campaign organized across all the villages that used multi-media approaches ^6^. Briefly, the ASHAs screened the adult population (age ≥18 years) using the Patient Health Questionnaire – 9 item (PHQ9) and Generalized Anxiety Disorder – 7 item (GAD7) to identify those suffering from stress, depression, increased suicide risk based on a score of ≥10 on either PHQ9/GAD7 or a score ≥1 on the suicidal ideation related question on PHQ9 ^7– 9^. Individuals screened positive were referred to the primary health care doctor who in turn used the mhGAP-IG modules to diagnose and treat the patients ^10^. Those with severe illnesses or with comorbid complications were referred to the psychiatrist at the district hospital. An algorithm based traffic light alerting system enabled the ASHAs to keep track of all patients screened positive by them and allowed them to follow-up and ensure better treatment adherence. Algorithm based voice messages sent via interactive voice recorded system facilitated the process of care by sending messages to patients, ASHAs and doctors about follow-up, monitoring, treatment.

### Formal evaluation of the project

A mixed methods pre-post evaluation was done using survey type questions, in-depth interviews and focus group discussions to assess the feasibility, acceptability and preliminary evidence regarding effectiveness. More details about the evaluation methodology can be found in a previous article ^11^.

### Identification of operational challenges

Besides the formal process evaluation, throughout the delivery of the study, there were some operational challenges that were identified and the research team had taken steps to overcome them. Some of the issues were identified during the evaluation too, but some others were corrected as an ongoing process throughout the study period. The operational issues were addressed through a number of strategies that we had put in place:

1. Regular meetings with the field staff and the project supervisors both before they left for field work in the morning and after they returned in the evening. Any operational challenges were discussed and troubleshooting was done to resolve them.
2. If the issue could not be resolved at the level of the project supervisors, it was discussed with research fellows during weekly meetings or over phone for more urgent matters and the issues were resolved.
3. If the research fellows were unable to resolve it, it was escalated to the Principal Investigator during weekly meetings.
4. During each interaction, notes were taken and the tasks identified to fix any operational issue was followed through by the line manager at each level.
5. Larger challenges at systems level were discussed during routine team meetings and solutions were identified and incorporated into the processes.
6. Besides meetings, all project supervisors and researchers made frequent visits to the sites to monitor activities and identify any unresolved issues. This included having discussions with community members, village leaders and village elders, government officials, and healthcare providers, as per need.
7. Discussions were also held with other researchers within the institute and senior faculty when needed to identify solutions to overcome challenges.

## Results

Some initial results from the SMART Mental Health Project have been published earlier ^5, 6, 11^. These results from the 30 tribal villages (Number of adults screened ~ 5000) showed that the intervention led to increased service use from a pre-intervention level of 0.8% to 12.6% at post-intervention. Depression and anxiety symptoms also reduced significantly, and the process evaluation could identify a number of barriers and facilitators. The results from the larger set of 12 non-tribal villages are being analysed.

Operationally, there were some challenges, especially related to internet connectivity, remote location of villages, monitoring of staff, and ensuring treatment access ( Table 1). Some steps were taken to overcome these operational challenges daily, and the key ones were: conducting an anti-stigma campaign; ensuring health camps in the villages; being pro-active and taking measures to get the buy-in from the community; engaging with village leaders when facing problems in the villages; trouble-shooting any issues faced by ASHAs, doctors or interviewers on a real-time basis; and monitoring data on a routine basis to check for inaccuracies.

**Table 1.** Key operational challenges faced in the SMART Mental Health project and strategies used to overcome them.

No	Category	Challenges faced	Strategies implemented to overcome the challenges
1	Staff recruitment	Difficulty in identifying people who had some basic knowledge about using tools based on mobile technology	• We reviewed our previous database of staff to identify anyone who was available for work • In some areas we had to ask local organizations to help identify individuals. • All staff were then provided with extensive training about how to use the mobile- technology based tools
2	Network Connectivity and availability of mobile phones for all patients	Due to the remoteness of some villages, internet connectivity was poor. This hampered the ability of data collected in the field being uploaded onto the servers. Often the families shared one mobile phone, and not necessarily would that phone be available with the patient at all times, making it difficult to send interactive voice response system (IVRS) based tailored messages to patients asking them to continue treatment	• We provided additional 3G hotspots at the primary health centres where field staff and ASHAs could come to upload data • We had used online apps to check for signal hotspots within each village and had informed staff about those locations • In-house training was given by the app developer to all field staff about how to use the SQLite browser and check pending status of any uploaded records • If any record could not be uploaded on the field even after this, such records were uploaded in-house at the project office after identifying and collecting the tablets • The field staff tried to identify the most suitable time of the day for sending messages to the patients and the IVRS was programmed to send messages keeping that in perspective
3	Distance of villages from primary health centres	The distance of some of the villages from the primary health centres prevented those in need from seeking care due to travel cost, wage loss, and loss of time	• Health camps were conducted in villages by the primary care doctor and such information about time and place were shared with ASHAs and the patients using interactive voice response messages and also through face-to-face meetings. The doctors were encouraged to go to these camps and see patients with mental disorders in addition to their other routine patients. • Coordination between the doctors and ASHAs and sharing of schedules was facilitated, at times, by the field staff
4	Stigma related to mental health	Lack of awareness and stigma related to mental health were reasons for people not identifying symptoms related to mental illness or seeking care	• An anti-stigma campaign using multi-media approaches was conducted prior to data collection to sensitize the population to mental health issues
5	Socio-cultural issues affecting data collection	Unavailability of household members, especially male members, for interviews at normal day times as they were often out in the fields or were at work At times the economically well-off community members refused to provide data believing that mental illness affected only the poor. This was at times also related to faulty caste perceptions that the backward castes were affected more with mental illness	• The field staff including ASHAs often went back to such people after they returned from the field or from their job, and also at times scheduled visits on weekends • The anti-stigma campaign helped to reduce some of the misconceptions, but at other times it needed more detailed one-to-one discussions about their misconceptions. • The buy-in from the local administration, which was sought at the outset, was also helpful as they and key village elders helped overcome some of the operational issues at village level
6	Availability of anti- depressants at primary health centres	Anti-depressants (fluoxetine/sertraline) were not made available at primary health centres. There were prolonged discussions with the regional government to provide these medicines to the targeted health centres, but bureaucratic hurdles prevented it from being functional eventually	• Those needing medicines had to be referred to the district hospitals to receive further care and free medicines. • Additionally, the government had established government medical stores aligned with the primary health centres which stocked generic and government-approved brands. This happened towards the end of the study. The field staff negotiated with those stores to stock cheaper brands of sertraline/fluoxetine, and the same information was shared with the primary care doctors to tell their patients
7	Ensuring follow-up and treatment adherence	Ensuring proper follow-up of those screened positive was an issue given the limited understanding of psychological treatment in the community	• The ASHAs were trained extensively about the importance of psychological treatments besides pharmacological treatments • The EDSS was developed in such a way that ASHAs had a traffic-light system algorithm on their tablets that alerted them every day to the status of all patients screened by them. This was used to prioritize follow-up by ASHAs with those diagnosed as suffering from mental disorders • At each follow-up the ASHAs were provided algorithm-based guide questions to specifically check about the treatment received with a purpose of ensuring treatment adherence. Specific focus was made on some of the psychological treatments (‘talk therapies’) prescribed by the primary care doctors such as identification of stressors, engaging with social contacts, participation in enjoyable activities. They also checked for use of medicines as prescribed, schedules for follow-up visits with doctors, and referral advices for specialist consultations
8	Monitoring of ASHAs and doctors	Since tablets and EDSS were new concepts for both ASHAs and doctors they needed proper training and monitoring	• Intensive training including hands-on experience was provided to both ASHAs and primary care doctors prior to the intervention on use of the tablet-based EDSS • Subsequently, close monitoring and trouble-shooting was done by the field staff on a need-to basis for some weeks which gradually reduced as ASHAs and doctors became more comfortable with the system
9	Site monitoring and data collection	Some selected villages were far from the field office, making it difficult for one person to monitor them regularly given limited transportation Data collection and monitoring the tribal villages were especially difficult given their remoteness Ensuring efficient utilization of staff time Continuous data monitoring had to be ensured	• Field supervisors were asked to take responsibility of extremely remote villages in turns so that one/two individuals did not feel burnt out • The tribal areas were so remote, that we had to arrange for our staff to stay at a rented apartment modified into a dormitory during the more intensive baseline data collection phase. Following that we retained only staff from the tribal villages for monitoring • The work plan of the field staff had to be monitored by supervisors and their daily routine carefully chalked out so that the time was used efficiently. This needed detailed discussions with the field staff and supervisors after each day’s work, and maintaining a staff scheduler and tracker of activities. Any problems were discussed and resolved as soon as possible via individual or group meetings before starting for work every day • Team leads were selected and they were entrusted with the responsibility of monitoring activities in the field • Data collected using the apps were checked routinely and often feedback was provided on a real-time basis as soon as the data was uploaded to ensure all problems were resolved as soon as possible.

Some operational issues continued to be unresolved satisfactorily and better strategies need to be implemented in the next stages of the project to overcome those. First, the supply of antidepressants in the primary health centres could not be ensured. Though generic stores had started towards the end of the study and patients were told to approach the nearest regular pharmacy to get the medicines, they often did not get them due to cost implications. Second, the patient often was not the key member in a house who owned a personal mobile. A shared phone was used in the household and often the interactive voice response system based messages sent to the patients to ensure treatment adherence did not reach them, even after the team tried to identify the most suitable time to send such messages, after speaking to the patients. Third, network connectivity continued to be an issue that hampered smooth functioning of data transfer, though it did not affect the delivery of any offline screening and diagnostic tools as part of the intervention.

## Discussion

The SMART Mental Health Project helped gather valuable data related to the feasibility of conducting such a study and understanding the processes better such that the next phase of the study, which involves conducting a cluster randomized controlled study, can be conducted more efficiently. Initial results suggested that the intervention had positive outcomes in the form of increased mental health services utilization, better depression and anxiety scores, and reduced stigma related to help-seeking. But, there were also process-related barriers and facilitators that need to be considered at subsequent phases of the study. Some of the processes outlined were assessed as part of the process evaluation ^11^. These included the methods used to deliver the anti-stigma campaign, organizing health camps, lack of anti-depressants in the primary care centres, non-availability of a personal mobile phone by all, and outcomes of the training imparted to interviewers and healthcare workers. This paper highlights some additional key operational issues that were identified and had to be managed on a regular basis to ensure delivery of the intervention as planned.

Both national and international policies advocate community based mental health services delivery and use of innovative techniques including digital solutions to maximize the reach and effectiveness of mental health services delivery models ^12, 13^. The SMART Mental Health project has established the acceptability and feasibility of delivering one such technology-based mental health services delivery model for a LMIC. This will need further testing and refining using more robust designs and will need to incorporate enhancements to make the mechanisms of service delivery more streamlined. The operational processes that needed fixing regularly or those that needed more systematic changes are seen by us as key to delivering such a project in similar settings, across the world, following suitable adaptations ^11^.

## Ethical standards

The authors assert that all procedures contributing to this work comply with the ethical standards of the relevant national and institutional committees on human experimentation (see previous publications for details ^5, 6, 11^) and with the Helsinki Declaration of 1975, as revised in 2008.

## Data availability

All data underlying the results are available as part of the article and no additional source data are required.
