# Supplementary material for: Operational challenges in conducting a community-based technology-enabled mental health services delivery model for rural India: Experiences from the SMART Mental Health Project
Source: Wellcome Open Res. 2018 Apr 18;3:43. [Version 1] doi: 10.12688/wellcomeopenres.14524.1 (PMC5941243; doi:10.12688/wellcomeopenres.14524.1)
Supplement: Peer review history

# Peer review: Referee response for version 1

**DOI:** 10.21956/wellcomeopenres.15811.r32937

**Published:** 2018-05-14

**Authors:** Atul Ambekar

**Affiliations:** 1 National Drug Dependence Treatment Centre (NDDTC), All India Institute of Medical Sciences (AIIMS), New Delhi, India

This is useful article which describes the operational challenges faced by the researchers while implementing an innovative, public-health-grounded intervention. Especially for LAMI countries such technology-based innovations are very important to address the service delivery gap. It is also notable that quite a few such innovations end up in a publication which highlights their 'effectiveness' while the operational issues - particularly important if such interventions were to be replicated elsewhere - remain largely undiscussed. It is creditable that authors list the operational issues they faced and also the strategies they employed to address these challenges. 

 I would have preferred some more discussion on which of the operational challenges were relatively easy to address and which required very specific strategies (which may be resource intensive). The basic premise of this intervention is to address mental health service delivery in low resource settings. If implementation of such resources need resource-intensive strategies, it would be difficult to replicate or scale up such interventions elsewhere. As specific examples, I wonder how feasible it would be to 'establish additional 3G hotspots' or 'training of field staff by the app developers', should such interventions be replicated at a large scale. Couple of sentences in the Discussion section can be devoted to this issue. Some estimates regarding enhanced resources (time and funds) required to meet the unforeseen operational challenges can also be presented in the Discussion section to increase the utility of this paper. 

 Overall, I welcome the publication of this paper.

I have read this submission. I believe that I have an appropriate level of expertise to confirm that it is of an acceptable scientific standard.

# Peer review: Referee response for version 1

**DOI:** 10.21956/wellcomeopenres.15811.r32935

**Published:** 2018-05-08

**Authors:** Gitanjali Narayanan, Ratnesh Chopra

**Affiliations:** 1 Department of Clinical Psychology, National Institute of Mental Health and Neuro Sciences (NIMHANS) , Bengaluru, India; 2 Center for Addiction Medicine, National Institute of Mental Health and Neuro Sciences (NIMHANS), Bengaluru, India; 3 Clinical Psychology, National Institute of Mental Health & Neuro Sciences, Bengaluru, India

The article is well conceptualized. It utilizes an innovative approach to address the existing treatment gap in mental health conditions in rural populations. As a part of a larger study, the paper provides a brief synopsis of the interventions being provided. The use of a technology enabled mental health service delivery program that involves training of lay counselors in different villages of Andhra Pradesh, India and the challenges experienced in implementation have been adequately discussed. It would be important to utilize the lessons learned from this phase of the study toward the subsequent part of the program.

We have read this submission. We believe that we have an appropriate level of expertise to confirm that it is of an acceptable scientific standard.

# Peer review: Referee response for version 1

**DOI:** 10.21956/wellcomeopenres.15811.r32939

**Published:** 2018-05-08

**Authors:** Julian Eaton

**Affiliations:** 1 Centre for Global Mental Health , London School of Hygiene and Tropical Medicine, London, UK

This succinct article outlines the findings related to operational challenges, as part of a wider process and preliminary outcome evaluation of a multifaceted, task shifting, stepped care intervention to address the treatment gap for mental disorders in rural villages in India. Understanding such challenges is an important part of further refining interventions, particularly at the stage of development here - in preparation for scaling up and larger-scale formal evaluation.  

 While the paper provides a fair amount of detail about the intervention, the details of the research evaluation methods and analysis used was so brief as to make it very difficult to judge rigour. Reference was made to an earlier paper, but it would be helpful to have more detail about how the results were reached. 

 The important point about decisions that need to be made by process evaluators about when to intervene to iteratively improve an intervention, and when to merely measure dispassionately, was not adequately developed. Such reflection is key to real-world evaluation and should have been discussed further (this point is made in the MRC Guidelines to Process Evaluation ^1^), particularly as this paper is itself about operational challenges and interventions to overcome them.  The researchers might clarify what rules/principles they used to decide when to adapt the intervention, and when not to. Was this, for example based on how far (time-wise) into the study they had reached, limiting intervention to early in a formative phase, or did they continue throughout, and in which case, how would they draw conclusions about the effects of changes to the implementation within the study period?

 The results - operational challenges identified - certainly rang true, and the responses were very pragmatic. It is not clear however, what the impact of the responses to challenges were (this does not seem to have been measured formally). 

 The brief discussion focuses in part on conclusions drawn from the wider study outside of this paper, though they do set the more restricted outcomes of this paper in context. Limitations to the paper were not discussed, but are detail is in linked papers.

 Overall, the issues raised in the paper are important, and it would be informative for any researchers or implementers undertaking to deliver well-evidenced services.

I have read this submission. I believe that I have an appropriate level of expertise to confirm that it is of an acceptable scientific standard.

## References

[1] MooreGFAudreySBarkerMBondLBonellCHardemanWMooreLO'CathainATinatiTWightDBairdJ: Process evaluation of complex interventions: Medical Research Council guidance. .2015;350:h1258 2579198310.1136/bmj.h1258PMC4366184
